# The *MaCreA* Gene Regulates Normal Conidiation and Microcycle Conidiation in *Metarhizium acridum*

**DOI:** 10.3389/fmicb.2019.01946

**Published:** 2019-08-21

**Authors:** Dongxu Song, Youhui Shi, HengQing Ji, Yuxian Xia, Guoxiong Peng

**Affiliations:** ^1^Genetic Engineering Research Center, School of Life Sciences, Chongqing University, Chongqing, China; ^2^Chongqing Engineering Research Center for Fungal Insecticide, Chongqing, China; ^3^Key Laboratory of Gene Function and Regulation Technologies Under Chongqing Municipal Education Commission, Chongqing, China; ^4^Chongqing Center for Disease Control and Prevention, Chongqing, China

**Keywords:** *creA*, normal conidiation, microcycle conidiation, conidiation pattern shift, *Metarhizium acridum*

## Abstract

As a C_2_H_2_ type zinc finger transcription factor, *CreA* is the key in Carbon Catabolism Repression (CCR) pathway, which negatively regulates the genes in carbon sources utilization. As conidiation in filamentous fungi is affected by nutritional conditions, *CreA* may contribute to fungal conidiation, which has been well studied in filamentous fungi, especially *Aspergillus* spp., but researches on entomopathogenic fungi are not enough. In this study, we found a homologous gene *MaCreA* in *Metarhizium acridum*, and the *MaCreA* deletion strain showed delayed conidiation, significant decrease in conidial yield, and 96.88% lower conidial production, when compared with the wild-type strain, and the normal conidiation and microcycle conidiation pattern shift was blocked. RT-qPCR showed that the transcription levels of the genes *FlbD* and *LaeA* (related to asexual development) were significantly altered, and those of most of the conidiation-related genes were higher in Δ*MaCreA* strain. The results of RNA-Seq revealed that *MaCreA* regulated the two conidiation patterns by mediating genes related to cell cycle, cell division, cell wall, and cell polarity. In conclusion, *CreA*, as a core regulatory gene in conidiation, provides new insight into the mechanism of conidiation in entomopathogenic fungi.

## Introduction

Conidia, as the asexual propagules in many filamentous fungi, are start and end of fungal lifecycle (Adams et al., 1998; Papagianni, 2004). They are the infective form of entomopathogenic fungi, essential for their pathogenicity (Schrank and Vainstein, 2010). In filamentous fungi, normal conidiation and microcycle conidiation are the two patterns of asexual conidiation (Zhang et al., 2010; Jung et al., 2014; Wang Z. et al., 2016). In normal conidiation, asexual conidia are produced at the top or sides of the hyphae in a subsequent budding-like process for proper vegetative growth, whereas microcycle conidiation occurs when fungi are subjected to unfavorable environmental conditions. As a survival mechanism, microcycle conidiation does not comprise the hyphae extension stage, and the conidia are directly generated from the germ tubes or conidial cells (Hanlin, 1994).

Asexual conidiation in the filamentous fungus *Aspergillus nidulans* has been well researched. When mycelium grow to a certain extent, under the condition of external stimuli, the hyphae starts to transform into aerial mycelium, and thick-walled foot cells are formed at the tip or side of the aerial mycelium. Following successive emergence of the foot cells, multinuclear conidiophores develop. The outer layer of conidiophores comprise vesicle, metulae, phialides, and aerial spores or conidia, which are formed sequentially (Borkovich and Ebbole, 2010; Park and Yu, 2012). Conidiation is regulated by a central regulatory pathway containing three genes *BrlA–AbaA–WetA* (Mirabito et al., 1989), and other transcription factors, such as *FluG*, which is an upstream gene of the entire pathway and a major activating protein of conidiation in *A. nidulans* (Lee and Adams, 1994b). In contrast, *SfgA*, which is downstream of *FluG* but upstream of *Flbs* (such as *FlbA*, *FlbB*, *FlbC*, *FlbD*) and *BrlA*, plays a negative regulatory role in conidiation (Seo et al., 2006). *FlbA* encodes a protein with a RGS domain, regulates the activity of the GTPase of *FadA*, a Gα protein (Lee and Adams, 1994a; Yang et al., 2012), and participates in mycelial development and asexual conidiation (Yu et al., 1996). *FlbB*, *FlbC*, *FlbD*, and *FlbE* are the activators of *brlA* (Etxebeste et al., 2009; Garzia et al., 2009; Kwon et al., 2010). While orthologs of *FlbA*, *FlbB*, *FlbC*, *FlbD*, and *FluG* have been found in *Metarhizium acridum*, those of *BrlA*, *AbaA*, and *WetA* have not been identified.

Although molecular studies on conidiation in *A. nidulans* have been conducted, the core regulatory pathway of *BrlA–AbaA–WetA* has been investigated only in certain *Aspergillus* spp., *Penicillium* spp., and *Talaromyces* spp. (López et al., 2018). Most of the entomopathogenic fungi, such as *Metarhizium* spp. and *Beauveria* spp., do not possess core regulatory pathway, implying that the central regulatory pathway is not common in all fungi. Moreover, in *M. acridum*, the core three genes in the central regulatory pathway have not been detected.

The phenomenon of microcycle conidiation is not commonly observed among fungi, and only more than 100 fungal species are currently known to exhibit this process (Jung et al., 2014). For instance, *Colletotrichum acutatum* has been noted to present obvious microcycle conidiation when incubated at 21.3–32.7°C for 12–36 h (Leandro et al., 2003), and *Penicillium variabile* has been reported to display microcycle conidiation in liquid culture (Zhelifonova et al., 2006). *Oidium longipes* is the first *powdery mildew* pathogen in which microcycle conidiation was observed when incubated on petunia leaves (Kiss et al., 2008). Besides, deletion of *WetA* gene has been noted to result in microcycle conidiation (Son et al., 2014). In *M. acridum*, microcycle conidiation has been reported to produce 4–5-fold higher conidia yield, with the generated conidia exhibiting increased heat resistance and trehalose level, but similar UV-B resistance and virulence, when compared with those produced by normal conidiation (Zhang et al., 2010). A recent study revealed that regulation of conidiation pattern in *M. acridum* is a complex process involving cell cycle and metabolism (Wang Z. et al., 2016). Nevertheless, the mechanism of microcycle conidiation remains unclear.

In the present study, we identified a homologous gene *CreA* in *M. acridum*, *MaCreA*, which was found to be a C_2_H_2_-type zinc finger transcription factor playing an important role in carbon catabolism repression (CCR) pathway in microorganisms (Dowzer and Kelly, 1991; Mathieu and Felenbok, 1994; Adnan et al., 2017). Besides, deletion of *MaCreA* caused delayed conidiation, significant decrease in conidial yield, and 98.88% lower conidial production, when compared with the wild-type strain, suggesting that *CreA* plays a crucial role in conidiation.

## Materials and Methods

### Strains and Growth Conditions

*Metarhizium acridum* strain CQMa102 was obtained from Chongqing Engineering Research Center for Fungal Insecticide and stored at China General Microbiological Culture Collection Center (CGMCC, No. 0877), and cultured on 1/4 SDAY medium (1% dextrose, 0.25% mycological peptone, 2% agar, and 0.5% yeast extract) at 28°C. *Escherichia coli* DH5α cells, used for DNA manipulations and transformations, were purchased from Bioground (China), and grown on Luria-Bertani (LB) medium at 37°C. *Agrobacterium tumefaciens* AGL-1 was purchased from Bioground (China) and propagated on LB medium at 28°C and used for fungal transformations.

### Construction of *MaCreA* Deletion Mutant and Complementation Mutant

The *MaCreA* disruption vector was constructed by homologous recombination based on the vector PK2-PB. About 1000-bp fragments upstream and downstream of the *MaCreA* open reading frame (ORF) were amplified from the WT genome by PCR, and primers used in this article is listed in Supplementary Table S1, then the two fragments were ligated to both the sides of the *bar* gene in PK2-PB to construct the knockout vector. The recombinant plasmid was transferred into the WT strain by *Agrobacterium*-mediated transformation. For the construction of complementation vector, the upstream 2000-bp fragment of the *MaCreA* ORF was amplified from the WT genome via PCR, ligated into the PK2-PB-SUR vector, and introduced into the knockout strain by *Agrobacterium*-mediated transformation. All the knockout and complementation strains were verified by Southern blot. The disruption and complementation of *MaCreA* were designed as shown in Supplementary Figures S1A,B.

### Measurements of Conidial Yield and Germination

To measure the conidial yield of the WT, Δ*MaCreA*, and complemented transformant (CP), 800 μL of 1/4 SDAY medium were first added to each well of the 24-cell plates. 2 μL of 10^6^ conidia/ml suspension of the WT, Δ*MaCreA*, and CP were inoculated into each well, and cultured at 28°C for 15 days. Three colonies of each strain were picked out every 2 days from the third day and suspended in sterile water for calculating conidial yield. The conidial germination rate of each strain was determined according to a previously described method (He and Xia, 2009). All experiments were performed in triplicate.

### Examination of Conidial Development

A total of 100 μL of 10^7^conidia/mL suspension of WT and Δ*MaCreA* were respectively spread on 1/4 SDAY and SYA (3% sucrose, 0.5% yeast extract, 0.3% NaNO_3_, 0.05% MgSO_4_, 0.05% KCl, 0.1% KH_2_PO_4_, 0.001% FeSO_4_, 0.001% MnSO_4_, and 2% agar) plates and incubated at 28°C. Subsequently, the fungal cells were detected under a digital light microscope (MOTIC, China) every 2 h from 12 h after incubation.

### Real-Time qPCR

For RNA extraction, the fungal cells were ground in liquid nitrogen, and the total RNA was isolated using Fungal RNA Kit (OMEGA) according to the manufacturer’s protocol. The quality of the extracted RNA was assessed by 2% agarose gel electrophoresis. To detect the expression levels of genes related to conidiation, reverse transcription of RNA was performed using PrimeScript^TM^ RT reagent Kit with gDNA Eraser. The genes *VeA, MAPK, NsdD, MedA, FlbA, LreA, VosA, Mmc, StuA, NsdC, FadA, CatA, FlbD, FlbB, SfgA, GanB, PKA, FluG, LreB*, and *LaeA* were chosen as target genes. qPCR was performed using SYBR^®^ Premix Ex Taq^TM^ (TaKaRa) according to the manufacturer’s protocol, and the relative expression levels of the target genes in the knockout strain and WT were computed by the 2^−ΔΔCt^ method (Livak and Schmittgen, 2001). All the experimental procedures were performed in triplicate.

### Digital Gene Expression Profiling and GO Analysis

RNA-Seq experiment based on Solexa sequencing was performed by Majorbio, Shanghai, China. The screening thresholds for differentially expressed genes (DEGs) are FDR ≤ 0.001 and Log_2_Ratio ≥ 1. All DEGs were mapped to the GO terms^1^ and KEGG pathways^2^. To verify the results of RNA-Seq, the transcript levels of 21 randomly selected DEGs were validated by RT-qPCR, and the results are shown in Supplementary Figure S2.

### RNA-Seq Data Accession

RNA-Seq data are deposited in the NCBI Sequence Read Archive (SRA) database, and the accession numbers are SRR9089704, SRR9089705, SRR9089702, SRR9089703, SRR9089700, SRR9089701, SRR9089698, SRR9089699, SRR9089696, SRR9089697, SRR9089706, and SRR9089707.

## Results

### Sequence Analysis of *MaCre*A

**FIGURE 1:**
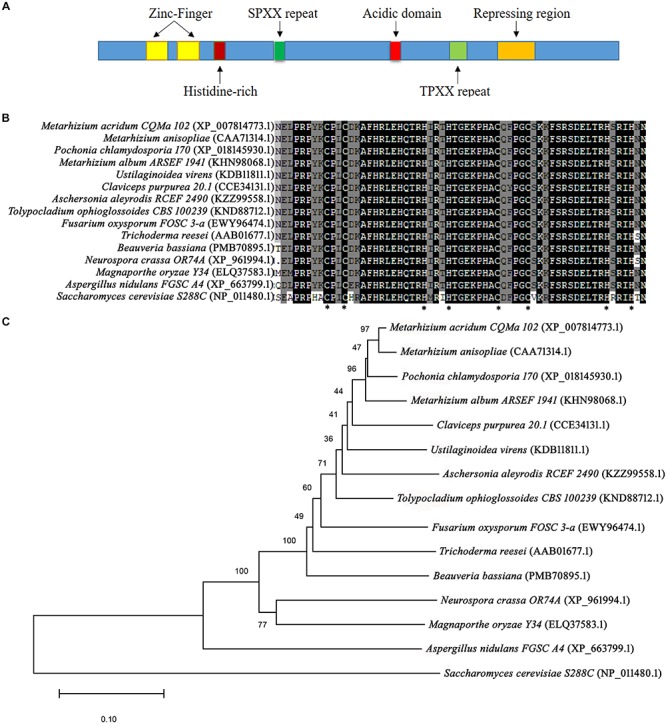
Sequences of *CreA* analysis. **(A)** Diagram of regions of *Metarhizium acridum CreA*. **(B)** Phylogenetic tree was reconstructed using the neighbor-joining method. **(C)** Comparison of C_2_H_2_ domain sequences of *MaCreA* with *CreA* homologs from other fungi.

Following BLAST search of the *M. acridum* genomic database, a *CreA* homologous gene was cloned and named as *MaCreA* (Accession number MK089826). The *MaCreA* comprised an ORF of 1131 bp and 376 amino acids, with predicted pI of 9.77 and molecular weight of 41 kDa^3^, and without signal peptide^4^. The gene exhibited two typical C_2_H_2_ zinc finger structures and other conserved domains (Figure 1A), similar to *BbcreA* in *Beauveria bassiana*. Phylogenetic analysis showed that *MaCreA* was closest to the near-origin *Metarhizium anisopliae CRR1* gene (Figure 1B). Homology analysis revealed that the similarity of *MaCreA* to *M. anisopliae* (CAA71314.1), *B. bassiana* (PMB70895.1), and *A. nidulans* (XP_663799.1) was 96.83, 77.46, and 53.57%, respectively (Figure 1C), and investigation of C_2_H_2_ zinc finger domains revealed 99% similarity in the selected fungi, indicating that *MaCreA* is a *CreA* homologous gene in *M. acridum.*

### *MaCreA* Is Required for Conidiation

**FIGURE 2:**
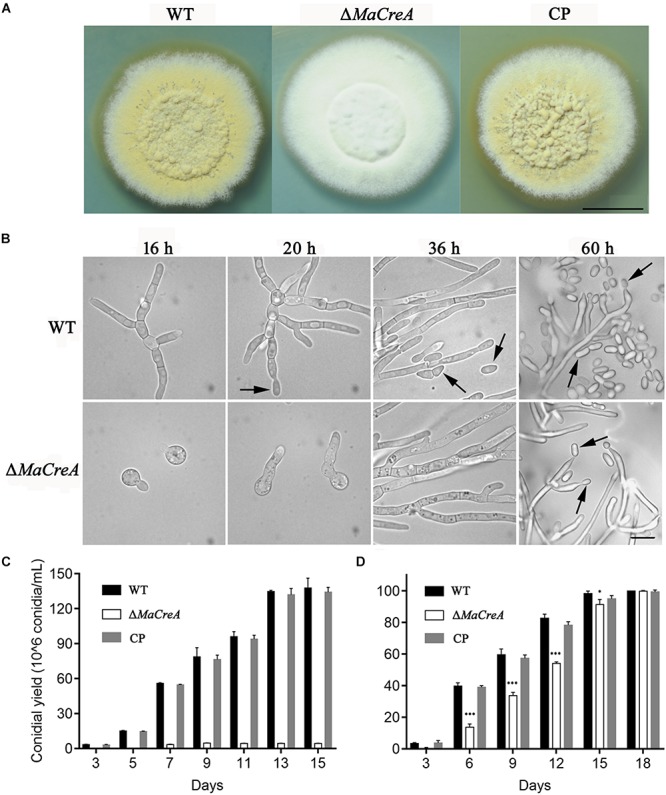
*MaCreA* deletion strain affects conidiation. **(A)** *MaCreA* deletion strain was grown for 7 days on 1/4 SDAY media, Scale bars indicate 1 cm. **(B)** Conidiation was postponed in *MaCreA* deletion strain on 1/4 SDAY. **(C)** Conidial yield of each stain on 1/4 SDAY media at 28°C, Scale bars indicate 10 μm, Black arrow: conidia. **(D)** Conidial germination of each stain on 1/4 SDAY media for 2, 4, 6, 8, 10, 12, and 14 h (*t*-text, ^∗^*p* < 0.05, ^∗∗^*p* < 0.01, ^∗∗∗^*p* < 0.001).

To exclude random insertion of genes into the genome, all the transformed *M. acridum* strains with deletion and complementation of *MaCreA* were finally verified by Southern blot analysis (Supplementary Figure S1C). With regard to the phenotype of the deletion strains, after 7-day growth on 1/4 SDAY medium, the WT and CP colonies became obviously tawny with mass production of conidia, while the colonies of the knockout strain were fluffy and white with strong mycelia (Figure 2A). To explore the effects of *MaCreA* on the conidiation process of *M. acridum*, morphogenesis of conidia was observed on 1/4 SDAY (rich medium) and SYA (poor medium) using a digital camera. The results showed that the WT produced conidiophores at 20 h after incubation on 1/4 SDAY medium, and the mature conidia were shed at 36 h; in contrast, Δ*MaCreA* presented obvious delay in conidiation, and the conidia were not observed until 60 h (Figure 2B). Evaluation of the conidial yields of all the three strains every 2 days revealed that the conidial yield of the knockout strain Δ*MaCreA* was significantly decreased, with 96.88% lower conidial production than that of the WT (Figure 2C). Moreover, the germination rate of the knockout strain was significantly lower than that of the WT and CP (Figure 2D).

### *MaCreA* Is Required for Conidiation Pattern Shift

**FIGURE 3:**
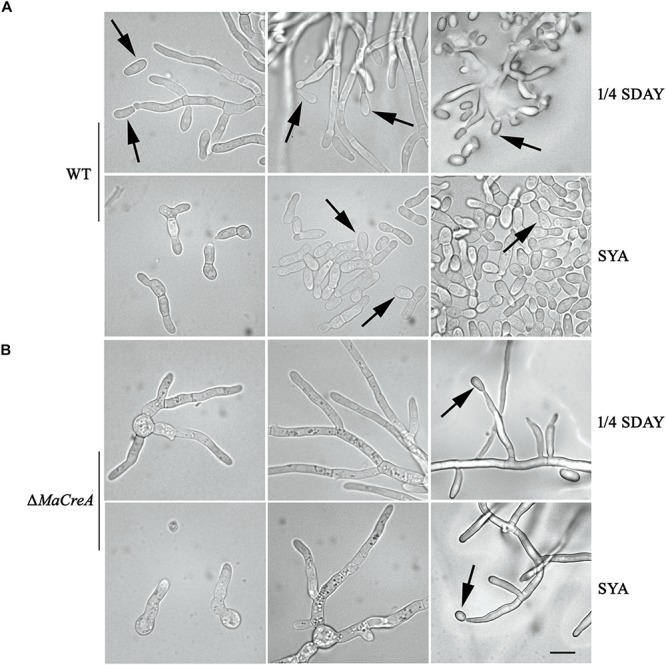
Conidiation pattern shift was lost in *MaCreA* deletion strain between 1/4 SDAY and SYA. Scale bars indicate 10 μm, Black arrow, conidia. **(A)** Conidiation of WT on 1/4 SDAY and SYA media. **(B)** Conidiation of Δ*MaCreA* on 1/4 SDAY and SYA media.

Two conidiation patterns are known in *M. acridum*, namely, normal conidiation and microcycle conidiation. In normal conidiation, conidia are produced at the top or sides of the hyphae, whereas in microcycle conidiation, conidia are directly generated from the germ tubes or conidial cells. In the present study, normal and microcycle conidiation patterns were observed in WT on 1/4 SDAY medium (rich medium) and SYA medium (poor medium), respectively (Figure 3A). However, after knocking out the *MaCreA* gene, the knockout strain exhibited a shift from microcycle conidiation to normal conidiation on SYA medium (Figure 3B), revealing that Δ*MaCreA* could not display microcycle conidiation under poor nutrient conditions (SYA medium), and that *MaCreA* gene played an important role in the shift of conidiation.

### Analysis of DEGs Involved in Conidiation in Fungal Cells Grown on Two Different Media

**FIGURE 4:**
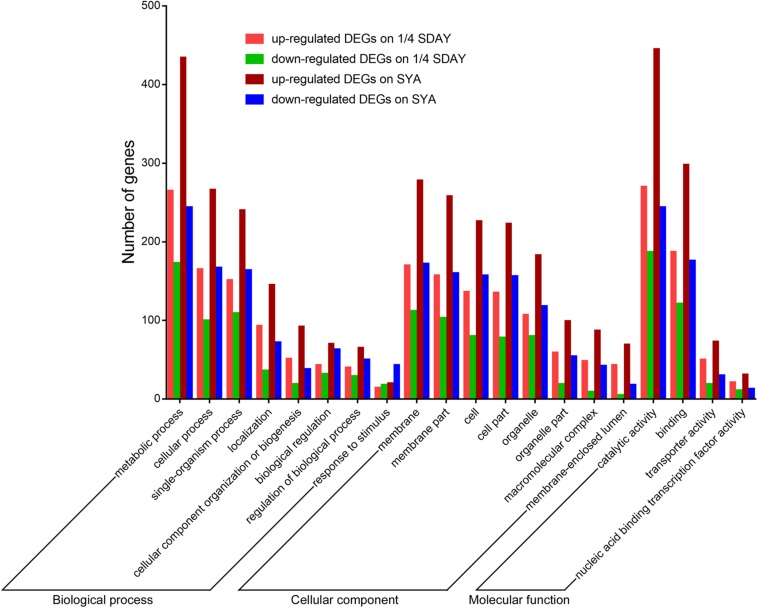
GO categories of DEGs on 1/4 SDAY and SYA. The lift *Y*-axis indicates the number of genes in a category; *X*-axis indicates the GO categories.

To determine the downstream genes and signaling pathways of *MaCreA* in conidiation, RNA-seq was used to identify significant up-regulated and down-regulated genes. The number of significant DEGs (twofold or greater, FDR ≤ 0.001) between Δ*MaCreA* and WT on 1/4 SDAY medium was 1099, among which 624 were up-regulated and 475 were down-regulated. However, 1743 DEGs (twofold or greater, FDR ≤ 0.001) were noted between Δ*MaCreA* and WT on SYA medium, of which 1039 were up-regulated and 704 were down-regulated. To investigate the conidiation pattern shift mechanisms involved in normal conidiation on 1/4 SDAY medium and microcycle conidiation on SYA medium, the differences and similarities between DEGs in fungal cells grown on the two media were examined. A total of 817 common DEGs in fungal cells grown on 1/4 SDAY and SYA media were noted, and 926 and 282 DEGs were detected in fungal cells grown on SYA medium and 1/4 SDAY medium, respectively. There are eight GO categories mainly enriched in biological process, eight in cellular component, and four in molecular function. Although the GO terms were similar in the two different media (Figure 4), 17 signaling pathways were only detected on SYA medium, suggesting that these pathways may respond to SYA medium, which are positive changes related to microcycle conidiation, such as lipid metabolism, carbohydrate metabolism, folding, sorting and degradation, signal transduction, and membrane transport (Supplementary Table S2).

**FIGURE 5:**
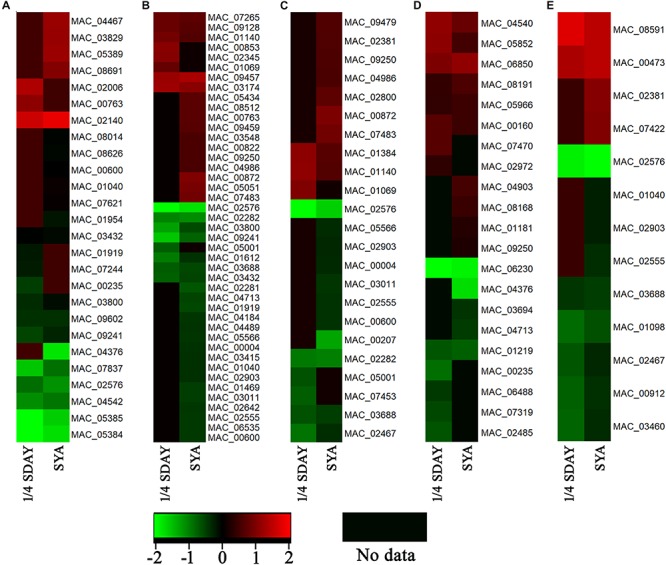
Expression profiling analysis of conidiation-relate genes in *CreA* deletion strain (Δ*MaCreA*) compared with wild-type strain (WT) in different culture states, namely, 1/4 SDAY and SYA. **(A)** genes involved in conidiation; **(B)** genes involved in cell cycle; **(C)** genes involved in cell division; **(D)** genes involved in cell wall; **(E)** genes involved in cell polarity.

To determine the DEGs involved in conidiation, DEGs in fungal cells grown on the two types of media were analyzed based on their GO annotations. Some genes related to conidiation were found, which were noted to be involved in the cell cycle, cell division, cell wall, and cell polarity. For example, the developmental protein FluG (MAC_08691), sporulation protein RMD8 (MAC_07621), APSES transcription factor (MAC_03829), putative UDP-glucose 4-epimerase (MAC_02140), and putative methyltransferase LaeA (MAC_03279), which are known to play important roles in conidiation in other fungi. Conidial pigment polyketide synthase PksP/Alb1 (MAC_05385), laccase (MAC_05384), laccase Lcc2 (MAC_02006), and Lcc5 (MAC_04467) are involved in conidial pigment synthesis. Pescadillo (MAC_07265), tyrosine-protein phosphatase CDC14 (MAC_03011), cell division cycle protein 123 (MAC_04986), and cell division control protein CDC48 (MAC_02555) are involved in cell proliferation. Mucin (MAC_01612), WASP-like protein las17 (MAC_00912), and p21 activated kinase-like protein (MAC_08591) are associated with cell polarity. In addition, a large number of cell wall related genes were also found, such as putative WSC domain protein (MAC_03694), cell wall protein (MAC_06850), and glycine-rich cell wall structural protein 1 (MAC_06488). These genes all play a role in conidiation of *M. acridium* (Supplementary Table S3). Analysis of the expression levels of these genes (Figure 5) revealed that these genes were down-regulated in the knockout strain compared with those in the WT, suggesting that the *CreA* gene had a significant effect on the conidiation process.

### *MaCreA* Affects Genes Related to Conidial Regulatory Pathway

**FIGURE 6:**
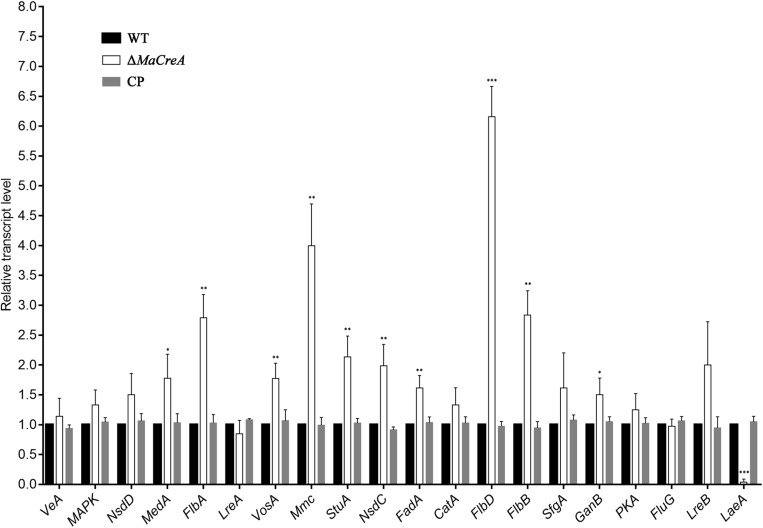
Relative expression of conidiation-related genes in Δ*MaCreA* (*t*-text, ^∗^*p* < 0.05, ^∗∗^*p* < 0.01, ^∗∗∗^*p* < 0.001).

To determine the relationship between *MaCreA* and conidiation pathway, the expression of conidiation-related homologous genes *VeA, MAPK, NsdD, MedA, FlbA, LreA, VosA, Mmc, StuA, NsdC, FadA, CatA, FlbD, FlbB, SfgA, GanB, PKA, FluG, LreB*, and *LaeA* in *M. acridum* was examined. To detect the expressions of conidiation-related genes in Δ*MaCreA* on 1/4 SDAY medium, RT-qPCR was performed, and the results showed that the expressions of *MedA*, *FlbA*, *VosA*, *StuA*, *NsdC*, *FadA*, *FlbD*, *FlbB*, and *GanB* in Δ*MaCreA* were higher than those in WT, with *FlbD* expression being significantly higher. In contrast, the expression of *LaeA* in Δ*MaCreA* was lower than those in WT, with significantly reduced. Furthermore, the expression of *Mmc*, which has been reported to be involved in microcycle conidiation (Liu et al., 2010), was also increased (Figure 6). It must be noted that three core genes, *BrlA*–*AbaA*–*WetA*, were not found in *M. acridum* as well as in other entomopathogenic fungi, but were only detected in *Aspergillus* and some *Penicillium* spp. (such as *Penicillium brasilianum*) (Supplementary Table S4). This indicated that the known asexual conidiation regulation pathway of *A. nidulans* was not common among fungi, and that different fungi may have unique conidiation regulation.

## Discussion

*CreA*, a gene involved in carbon sources utilization, plays a pivotal role in the CCR pathway with a wide range of regulation. This gene is a C_2_H_2_ transcription factor and its zinc finger structure is highly conserved from yeasts to human pathogens, implying that *CreA* plays an important role among different species. In *Aspergillus niger*, the feruloyl esterase gene *faeA*, alpha-glucuronidase gene *aguA*, endoxylanase gene *xlnB*, and beta-xylosidase gene *xlnD* in xylose metabolism have been noted to be repressed by *CreA* (de Vries et al., 1999). In *A. nidulans*, *CreA* has been observed to mediate indirect repression of *xlnR*, which controls the production of xylanolytic enzymes (Tamayo et al., 2008). In *B. bassiana*, deletion of *BbcreA* has been demonstrated to result in nutrient toxicity, conidial yield reduction, and defects in virulence (Luo et al., 2014).

While most of the previous studies on *CreA* had focused on carbon sources metabolism, only a few works had examined the effects of this gene on conidiation in fungi. In the present study, the colonies of Δ*MaCreA* were noted to be fluffy and white, with strong mycelium, obviously delayed conidiation, and severe decrease in conidial yield. In addition, *CreA* deletion strain of the *Magnaporthe oryzae* and *Penicillium oxalicum* also demonstrated a decrease in conidial yield (Cao et al., 2016; Zhang et al., 2016). The quantitative PCR (qPCR) experiments showed that the expressions of *LaeA* decreased significantly in Δ*MaCreA* while the *Flb*s (*FlbA*, *FlbB*, and *FlbD*) increased significantly, suggesting that *CreA* positively regulates *LaeA* and negatively regulates *Flb*s. The *Flb* genes have homologous genes in entomopathogenic fungus *B. bassiana*, and all of them are involved in the conidiation process (Chu et al., 2017). *MoRgs1*, as a homologous gene of *FlbA*, has a positive role in conidiation in *M. oryzae* (Zhang et al., 2011). *Chlae1*, a homologous gene of *LaeA*, is present in plant pathogenic fungi *Cochliobolus heterostrophus*, which clearly affects its asexual development (Wu et al., 2012). Moreover, *StuA* has been recently identified to regulate conidiation in *M. robertsii* (Yang et al., 2018). The expressions of these conidiation related genes had been altered in *CreA* deletion strain, Therefore, it is reasonable to speculate that *CreA* is involved in the conidiation regulatory pathway of *M. acridium*.

While asexual conidiation of filamentous fungi has been extensively studied, systematic investigations have been conducted only on *Aspergillus* spp., especially *A. nidulans*, in which the functions of genes for asexual conidiation have been elucidated. In the present study, analysis of the genomes of entomopathogenic fungi for homologs of asexual conidiation genes showed that most of the fungi did not possess related core homologous genes, consistent with that reported in previous study (López et al., 2018). Similarly, a recent research on *Zymoseptoria tritici* showed that asexual conidiation observed in *A. nidulans* is only partially relevant in *Z. tritici*, suggesting the presence of uncharacterized genes that control asexual conidiation in this pathogen (Tiley et al., 2018). and indicating that entomopathogenic fungi have their own unique asexual conidiation system. In nature, entomopathogenic fungi have evolved highly diversified survival strategies with co-evolutionary relationships between pathogens and insect hosts (Gao et al., 2011; Wang J.B. et al., 2016; Wang and Wang, 2017), implying rapid changes in their genome sequences to accommodate their own survival. Therefore, asexual conidiation in entomopathogenic fungi also vary.

In the present study, on SYA medium, conidiation was significantly delayed and microcycle conidiation was absent in Δ*MaCreA*, indicating that *MaCreA* regulates the shift of the two conidiation patterns. The researches on the mechanism of microcycle conidiation is still scarce. Recently, studies about microcycle conidiation mostly focus on the phenotypic observation in different fungi (Souza-Paccola et al., 2015; van Heerden et al., 2016; Rosli et al., 2018). In the preliminary work in our lab, we found that normal conidiation and microcycle conidiation are involved in lipid metabolism, sugar chain biosynthesis, and metabolism and translation (Wang Z. et al., 2016). Nevertheless, the mechanism of the shifts of the two conidiation patterns remains unclear. Some DEGs are involved in the conidiation process in filamentous fungi, such as fluG, sporulation protein RMD8, APSES transcription factor, and putative UDP-glucose 4-epimerase (Seo et al., 2006; El-Ganiny et al., 2010; Gao et al., 2011; Yang et al., 2018). There are four genes involved in pigmentation synthesis, such as *PksP*/*Alb1*, laccase, and laccase *Lcc2* and *Lcc5* (Langfelder et al., 1998; Rivera-Hoyos et al., 2013). After conidia germination, changes in cell polarity occur (Momany, 2002; Harris, 2006). DEGs of Mucin, WASP-like protein las17, and p21 activated kinase-like protein are involved in cell polarity (Goehring et al., 2003; Pitoniak et al., 2009; Robertson et al., 2009). Therefore, we have reason to conclude that conidiation process also related to polarity changes, when the conidiation process begins, the hyphal polarity maintenance state will converted into isotropic expansion of the daughter conidial cells. Together, these genes are downstream of *MaCreA* and affect the conidiaion process of *M. acridium*.

## Conclusion

In general, the deletion strain Δ*MaCreA* exhibited a significant decrease in conidiation yield, indicating that *MaCreA* played an important role in the conidiation process in the entomopathogenic fungus *M. acridum* and was a core conidiation regulatory gene. RT-qPCR revealed that *MaCreA* had certain effections on other known conidiation regulatory genes. High-throughput sequencing results confirmed that deletion of *MaCreA* altered the expressions of a large number of genes involved in cell cycle, cell division, conidiation, and cell polarity, demonstrating that this gene had a significant function in the process of conidiation. Thus, these results indicated that *MaCreA* was a core conidiation regulatory gene in *M. acridum*, providing a new insight into the process of conidiation in entomopathogenic fungi.

## Data Availability

The datasets generated for this study can be accessed from the RNA-Seq data, are deposited in the NCBI Sequence Read Archive (SRA) database, and the accession numbers are SRR9089704, SRR9089705, SRR9089702, SRR9089703, SRR9089700, SRR9089701, SRR9089698, SRR9089699, SRR9089696, SRR9089697, SRR9089706, and SRR9089707.

## Author Contributions

DS and YS contributed equally to this work. HJ analyzed the RNA-Seq data. All authors read and approved the final manuscript.

## Conflict of Interest Statement

The authors declare that the research was conducted in the absence of any commercial or financial relationships that could be construed as a potential conflict of interest.

## Footnotes

1 http://www.geneontology.org/

2 https://www.kegg.jp/

3 https://web.expasy.org/protparam/

4 http://www.cbs.dtu.dk/services/SignalP/
