# Supplementary material for: The MaCreA Gene Regulates Normal Conidiation and Microcycle Conidiation in Metarhizium acridum
Source: Front Microbiol. 2019 Aug 21;10:1946. doi: 10.3389/fmicb.2019.01946 (PMC6713048; doi:10.3389/fmicb.2019.01946)
Supplement: Supplementary file 1 [file Data_Sheet_1.pdf]

Supplementary Material for ***Frontiers in Microbiology***

The *MaCreA* gene regulates normal conidiation and microcycle conidiation in *Metarhizium acridum*

Dongxu Song[1,2,3#] · Youhui Shi[1,2,3#] · HengQing Ji[4] ·Yuxian Xia[1,2,3]* · Guoxiong Peng[1,2,3]*

[1]Genetic Engineering Research Center, School of Life Sciences, Chongqing University, Chongqing 401331, People's of Republic of China
[2]Chongqing Engineering Research Center for Fungal Insecticide, Chongqing 401331, People's of Republic of China
[3]Key Laboratory of Gene Function and Regulation Technologies under Chongqing Municipal Education Commission, Chongqing 401331, People's of Republic of China
[4]Chongqing Center for Disease Control and Prevention

#Both authors contributed equally to this work.
*Correspondence to: Guoxiong Peng, E-mail: gxpeng@cqu.edu.cn; ORCID: 0000-0003-2443-8691; Tel.: +86-185 8463 5550 and Yuxian Xia, E-mail: yuxianxia@cqu.edu.cn; ORCID:0000-0001-5551-703X; Tel.: +86-185 8463 5550

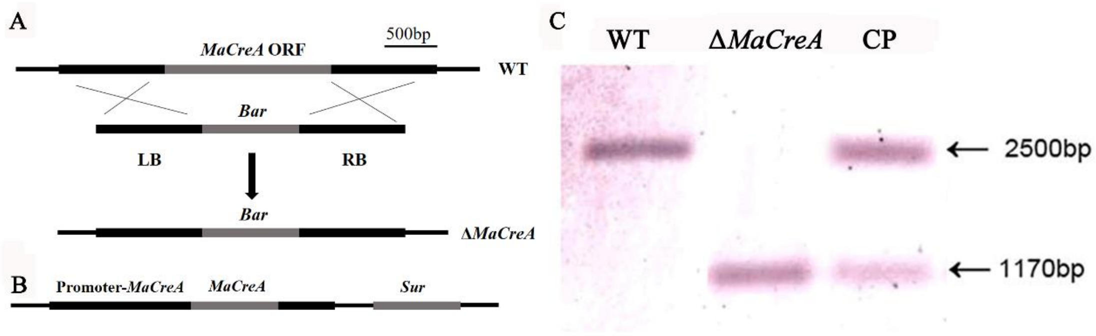

Figure S1 Disruption and complementation of *MaCreA*. **(A)** *MaCreA* was disrupted by homologous recombination. **(B)** Design of the *MaCreA* complementation plasmid. **(C)** Validation of transformants by Southern blotting.

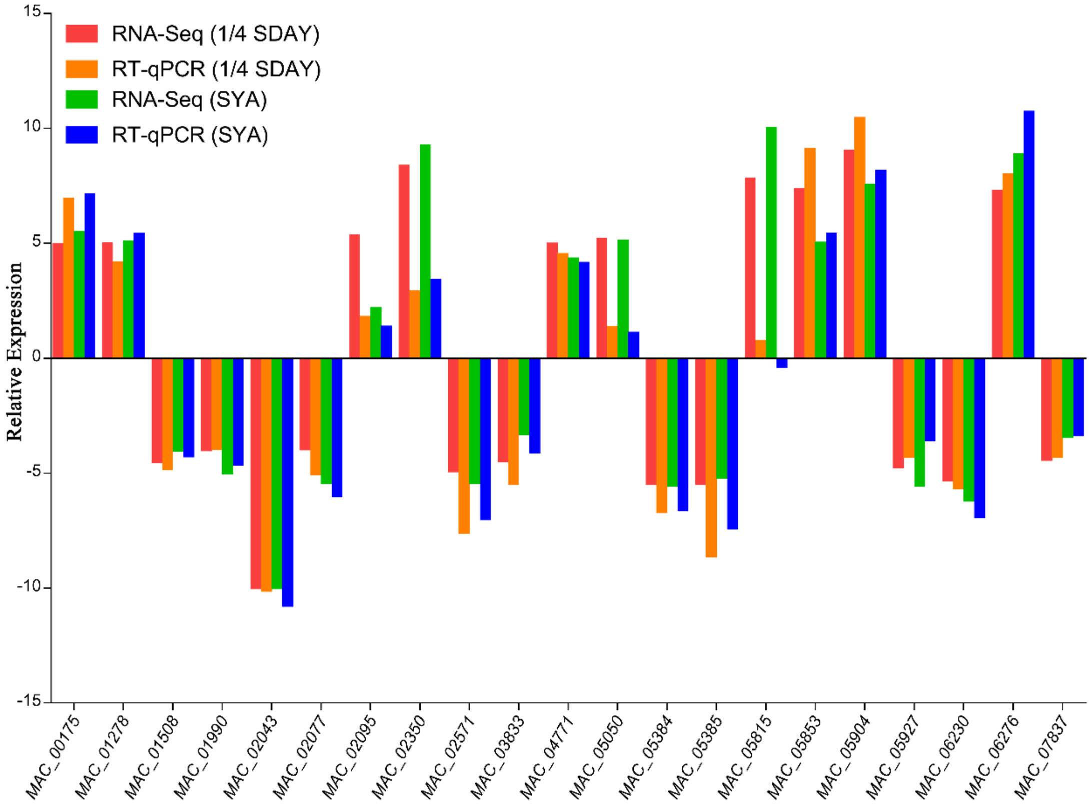

Figure S2 The verifications of the transcript levels of 21 different expression genes in RNA-Seq.

Table S1. Paired primers used in this paper

Primer	Paired sequences (5′–3′)	Purpose
MaCreA-LF	CCCAAGCTTGCTTGAACGGAGTACGGAGTA	Cloning *CreA* 5′-end
MaCreA-LR	CGCGGATCCGTCTCACCTCCTCACTCCTTC	Cloning *CreA* 5′-end
MaCreA-RF	GCGATATC TCCGCAACTTGTCGCTTCA	Cloning *CreA* 3′-end
MaCreA-RR	CCGGAATTCTTAGACTGGCTGACGCATACAT	Cloning *CreA* 3′-end
CP-*MaCreA*-F	GACGGCCAGTGCCAAGCTTGAGCATGACCATTGTGTGC	Cloning full-length *CreA*
CP-*MaCreA*-R	TGCTCTCACGTCGACGAT AACCCGGTCCATCAGGTCGC	Cloning full-length *CreA*
sb*MaCreA*-F	GCTTGAACGGAGTACGGAGTA	Southern probe for *CreA*
sb*MaCreA*-R	GTCTCACCTCCTCACTCCTTC	Southern probe for *CreA*
MaFlbA-F	AATACACCTTCCTCTCAGA	RT-qPCR detecting *FlbA*
MaFlbA-R	TCGTCGTGGTAACAATTC	RT-qPCR detecting *FlbA*
MaFlbB-F	ATAAGCAAGGCTGATCTG	RT-qPCR detecting *FlbB*
MaFlbB-R	GAATCGTGGATGACTCAA	RT-qPCR detecting *FlbB*
MaFlbC-F	CTTATGGGTTTCCTTCTA	RT-qPCR detecting *FlbC*
MaFlbC-R	GCTGAAGAGTAATCTGATA	RT-qPCR detecting *FlbC*
MaCreA-F	GGTTCAGGTTCAGTTCCA	RT-qPCR detecting *CreA*
MaCreA-R	GAGCAGTGTTGGTCTCAA	RT-qPCR detecting *CreA*
MaFluG-F	AAAGACAGTCAGTAAATG	RT-qPCR detecting *FluG*
MaFluG-R	CAGTGTGGAATTGAATAG	RT-qPCR detecting *FluG*
MaSfgA-F	AGACAGATATAAGCAAGT	RT-qPCR detecting *SfgA*
MaSfgA-R	TGGTGTAATCGTAGATAA	RT-qPCR detecting *SfgA*
VeA-F	TTCCGTCAGTCAAGCAT	RT-qPCR detecting *VeA*
VeA-R	TGGCAGTAATGGCGTAA	RT-qPCR detecting *VeA*
MAPK-F	AAGATGTTGTAGGAGAAGGT	RT-qPCR detecting *MAPK*
MAPK-R	TGATTGAAGTAGCGAAGGA	RT-qPCR detecting *MAPK*
NsdD-F	ATGCTCACGCTTCCAAT	RT-qPCR detecting *NsdD*
NsdD-R	GTAGTTCCACGGCTCAA	RT-qPCR detecting *NsdD*
MedA-F	ACTACCTACAGAAGCAACA	RT-qPCR detecting *MedA*
MedA-R	GCCGTCCACTACTCATT	RT-qPCR detecting *MedA*
FlbA-F	CCACCTTCACAGCCATA	RT-qPCR detecting *FlbA*
FlbA-R	CGGTCATCGTCAGTCAT	RT-qPCR detecting *FlbA*
LreA-F	AGCCTCAACATCATCTCAT	RT-qPCR detecting *LreA*
LreA-R	CCTATTCACCTTCTCCATCT	RT-qPCR detecting *LreA*
VosA-F	GACAAGCGGACTGATGG	RT-qPCR detecting *VosA*
VosA-R	GATGGATAGATGATGCGAGAG	RT-qPCR detecting *VosA*
Mmc-F	CTGAGGTCGTTGAGAAGT	RT-qPCR detecting *Mmc*
Mmc-R	GACAGGCTTGGTGATAGT	RT-qPCR detecting *Mmc*
StuA-F	GCTCCGCATCAGTCATA	RT-qPCR detecting *StuA*
StuA-R	AGGCACCATTCATCAGT	RT-qPCR detecting *StuA*
VelB-F	ATCCACAACCTCATCAACT	RT-qPCR detecting *VelB*
VelB-R	CCAGTCGCCTCATCAAT	RT-qPCR detecting *VelB*
NsdC-F	CTACGATGGTGTGGAAGATG	RT-qPCR detecting *NsdC*
NsdC-R	AGGATATGTGCGAGAGTGT	RT-qPCR detecting *NsdC*

FadA-F	TCAAGGTGGTTCATCAAGA	RT-qPCR detecting *FadA*
FadA-R	CCGCAGCATAGTCATCA	RT-qPCR detecting *FadA*
CatA-F	GTGAGCAGCGACAAGAC	RT-qPCR detecting *CatA*
CatA-R	GAGAAGCGGACGAAGAC	RT-qPCR detecting *CatA*
FlbD-F	CTGCCTCGTATTAGTCCAA	RT-qPCR detecting *FlbD*
FlbD-R	TTCCTTCCTCCTCCTTCT	RT-qPCR detecting *FlbD*
FlbB-F	TGTCGTCTTCATTATCTCCT	RT-qPCR detecting *FlbB*
FlbB-R	GTTGTGCTTGTCGGTTC	RT-qPCR detecting *FlbB*
SfgA-F	AAGCAAGTTCGTCAAGATG	RT-qPCR detecting *SfgA*
SfgA-R	GGCAGTCAGTGTCTCAA	RT-qPCR detecting *SfgA*
GanB-F	GAGGCGGAACAGAAGAA	RT-qPCR detecting *GanB*
GanB-R	GCTTGACGATGGTAGACT	RT-qPCR detecting *GanB*
PKA-F	CGAAGCATAACCAACGATT	RT-qPCR detecting *PKA*
PKA-R	TCCTTAGCAGTGAGAATAGC	RT-qPCR detecting *PKA*
FluG-F	CGAACAGGACTTGACATTG	RT-qPCR detecting *FluG*
FluG-R	GACGGTGGACGATGAAT	RT-qPCR detecting *FluG*
FlbC-F	GGTCATCATCAACATCTCCTT	RT-qPCR detecting *FlbC*
FlbC-R	GAAGTCGCATAGGTAGTATCAC	RT-qPCR detecting *FlbC*
LreB-F	CAGGCGAGAAGAAGAAGA	RT-qPCR detecting *LreB*
LreB-R	TAGCGTCACAGTCAGTAAG	RT-qPCR detecting *LreB*
LaeA-F	TTAGATGGACTGACGACGATA	RT-qPCR detecting *LaeA*
LaeA-R	GGTTGCGGTAGTTGTAGATG	RT-qPCR detecting *LaeA*
Gapdh-F	GACTGCCCGCATTGAGAAG	RT-qPCR detecting *Gapdh*
Gapdh-R	AGATGGAGGAGTGGGTGTTG	RT-qPCR detecting *Gapdh*
qF-MAC_00175	CGAGGAGGGCAATGACAA	RT-qPCR verifing RNA-Seq
qR-MAC_00175	CCACCAGTATGACGAAGGAT	RT-qPCR verifing RNA-Seq
qF-MAC_01278	CCTCTGATGCCACTCTACTC	RT-qPCR verifing RNA-Seq
qR-MAC_01278	TCAACATAACGAAGCCAACTG	RT-qPCR verifing RNA-Seq
qF-MAC_01508	CCGCCAAGAAGATAGAGGAT	RT-qPCR verifing RNA-Seq
qR-MAC_01508	GCAAGGAACAGAAAGGTAAACA	RT-qPCR verifing RNA-Seq
qF-MAC_01990	ACCTCTCGTCCTACCCAA	RT-qPCR verifing RNA-Seq
qR-MAC_01990	TCCAGTTCCAGCCACAAT	RT-qPCR verifing RNA-Seq
qF-MAC_02043	CCAGCACCCAAGTCATCA	RT-qPCR verifing RNA-Seq
qR-MAC_02043	GCAGTAGTCACCGTAGCA	RT-qPCR verifing RNA-Seq
qF-MAC_02077	GGTTCGTGTCAAGTGTCTATTAC	RT-qPCR verifing RNA-Seq
qR-MAC_02077	GTCCAGGCAAGGGTGTAT	RT-qPCR verifing RNA-Seq
qF-MAC_02095	CTTCCGCTACGCACAAATC	RT-qPCR verifing RNA-Seq
qR-MAC_02095	TTGGCTCCGACTGGTAATAG	RT-qPCR verifing RNA-Seq
qF-MAC_02350	CGTAATGCTGGTGCTTTCC	RT-qPCR verifing RNA-Seq
qR-MAC_02350	GTGCCTTGGTCTTGTTGTC	RT-qPCR verifing RNA-Seq
qF-MAC_02571	CCGTTGATGAATGGAATGCT	RT-qPCR verifing RNA-Seq
qR-MAC_02571	TGTAAGTGGTGCCGTTGT	RT-qPCR verifing RNA-Seq
qF-MAC_03833	GTCCGAGAAGTCCGAGAAG	RT-qPCR verifing RNA-Seq
qR-MAC_03833	GGTGGCTATCCTTGTCCTT	RT-qPCR verifing RNA-Seq

qF-MAC_04771	TACTACGATGTTGACCTTGTGA	RT-qPCR verifing RNA-Seq
qR-MAC_04771	CTTTCTGGCTTGCGATGG	RT-qPCR verifing RNA-Seq
qF-MAC_05050	GAGGCAGGATGTGTTGGA	RT-qPCR verifing RNA-Seq
qR-MAC_05050	GGTAGAGTTGATAGAGGTTGTGA	RT-qPCR verifing RNA-Seq
qF-MAC_05384	AGTTTGTCTCGGCAGTCAT	RT-qPCR verifing RNA-Seq
qR-MAC_05384	CGCTCGTGTCCTTCGTAA	RT-qPCR verifing RNA-Seq
qF-MAC_05385	ACGGTATGACAAGCGATGA	RT-qPCR verifing RNA-Seq
qR-MAC_05385	GCAAGCCAAGTGAATAGCC	RT-qPCR verifing RNA-Seq
qF-MAC_05815	TTCAAGGAGGAGTCGTCATC	RT-qPCR verifing RNA-Seq
qR-MAC_05815	AAGCAGCGTAGGAGAGTG	RT-qPCR verifing RNA-Seq
qF-MAC_05853	TACCGCCGTCAACAACTA	RT-qPCR verifing RNA-Seq
qR-MAC_05853	GGAGAGGGAAGCATCAATAGA	RT-qPCR verifing RNA-Seq
qF-MAC_05904	CTTGCTCATCTCCGTTGC	RT-qPCR verifing RNA-Seq
qR-MAC_05904	CATTGCCTTGTTGACCTCTT	RT-qPCR verifing RNA-Seq
qF-MAC_05927	AGGTTGAGGAAGAGGATGAC	RT-qPCR verifing RNA-Seq
qR-MAC_05927	CCGTGGCGATGATACAGA	RT-qPCR verifing RNA-Seq
qF-MAC_06230	GTCCTGTGGTGTCGTGAA	RT-qPCR verifing RNA-Seq
qR-MAC_06230	CTGGTATGAAGTGTGGTAGTAGA	RT-qPCR verifing RNA-Seq
qF-MAC_06276	GCGACCTGACCTATGTTGA	RT-qPCR verifing RNA-Seq
qR-MAC_06276	ACCGATGGAATGGCTTGA	RT-qPCR verifing RNA-Seq
qF-MAC_07837	ACTATGCCATTCCGTCCAA	RT-qPCR verifing RNA-Seq
qR-MAC_07837	AACTCTGTGTCTCGTGCTT	RT-qPCR verifing RNA-Seq

Table S2. Pathways respond to microcycle conidiation.

First Category	Second Category	Description
Metabolism	Lipid metabolism	Synthesis and degradation of ketone bodies
Metabolism	Biosynthesis of other secondary metabolites	Monobactam biosynthesis
Metabolism	Carbohydrate metabolism	C5-Branched dibasic acid metabolism
Metabolism	Glycan biosynthesis and metabolism	N-Glycan biosynthesis
Metabolism	Glycan biosynthesis and metabolism	Other glycan degradation
Metabolism	Metabolism of other amino acids	Selenocompound metabolism
Metabolism	Lipid metabolism	alpha-Linolenic acid metabolism
Metabolism	Metabolism of terpenoids and polyketides	Terpenoid backbone biosynthesis
Metabolism	Lipid metabolism	Biosynthesis of unsaturated fatty acids
Genetic Information Processing	Folding, sorting and degradation	Sulfur relay system

Genetic Information Processing	Folding, sorting and degradation	Protein processing in endoplasmic reticulum
Genetic Information Processing	Replication and repair	Non-homologous end-joining
Environmental Information Processing	Signal transduction	Hippo signaling pathway -multiple species
Environmental Information Processing	Signal transduction	Phosphatidylinositol signaling system
Environmental Information Processing	Membrane transport	ABC transporters
Cellular Processes	Transport and catabolism	Phagosome
Human Diseases	Endocrine and metabolic diseases	AGE-RAGE signaling pathway in diabetic complications

Table S3. Functions of conidiation-relate genes

Function	Gene	Name	On SYA or 1/4 SDAY
Cell cycle	MAC_04489	BTB/POZ domain protein	1/4 SDAY
	MAC_09459	CAMK family protein kinase	SYA
	MAC_09128	protein tyrosine phosphatase Pps1, putative	SYA, 1/4 SDAY
	MAC_03548	nuclear envelope protein	SYA
	MAC_07265	pescadillo	SYA, 1/4 SDAY
	MAC_01469	DNA-repair protein rad2	SYA
	MAC_04184	cyclin-like protein (Clg1), putative	SYA
	MAC_02281	serine/threonine-protein kinase prp4	SYA
	MAC_03415	putative kinetochore protein ndc-80	SYA
Cell cycle & cell division	MAC_02282	Ran1-like protein kinase	SYA, 1/4 SDAY
	MAC_01069	DNA mismatch repair protein msh3	1/4 SDAY
	MAC_07483	methyltransferase	SYA
	MAC_01140	gamma-tubulin	SYA, 1/4 SDAY
	MAC_03011	tyrosine-protein phosphatase CDC14	SYA
	MAC_04986	cell division cycle protein 123	SYA
Cell cycle & cell division & cell polarity	MAC_03688	calponin	SYA, 1/4 SDAY
	MAC_02903	putative maintenance of ploidy protein mob1	SYA
	MAC_02555	cell division control protein Cdc48	SYA
	MAC_05566	protein hob3	SYA
Cell cycle & cell polarity & conidiation	MAC_01040	Noc1p protein, putative	SYA
Cell cycle & conidiation	MAC_01919	trehalose-6-phosphate synthase 1	SYA
	MAC_09241	NIF domain protein	SYA, 1/4 SDAY
	MAC_03800	sphingolipid long chain base-responsive protein PIL1	SYA, 1/4 SDAY
	MAC_00763	ribosome biogenesis GTPase Lsg1, putative	SYA

cell division	MAC_07453	SNARE-dependent exocytosis protein (Sro7), putative	1/4 SDAY
	MAC_02800	protein prenyltransferase alpha subunit repeat protein	SYA
	MAC_09479	U3 snoRNP protein	SYA
cell division & cell polarity	MAC_02467	pheromone receptor	SYA, 1/4 SDAY
cell polarity	MAC_01098	putative BAR adaptor protein	SYA, 1/4 SDAY
	MAC_01612	mucin, putative	SYA, 1/4 SDAY
	MAC_03460	PH domain protein	SYA, 1/4 SDAY
	MAC_00912	WASP-like pretein las17p	SYA, 1/4 SDAY
	MAC_07422	cell polarity protein, putative	SYA
	MAC_08591	p21 activated kinase-like protein	SYA, 1/4 SDAY
cell wall	MAC_01219	glycoside hydrolase family 24 protein	SYA, 1/4 SDAY
	MAC_00235	phosphoinositide 3-phosphate phosphatase	1/4 SDAY
	MAC_02485	N,O-diacetyl muramidase, putative	1/4 SDAY
	MAC_02972	subtilisin-like protease	1/4 SDAY
	MAC_04376	hydrophobin	SYA
	MAC_04713	phosphatidylserine synthase	SYA
	MAC_07470	subtilisin-like serine protease PR1C	1/4 SDAY
	MAC_08191	ribose-phosphate pyrophosphokinase	SYA, 1/4 SDAY
	MAC_03694	WSC domain protein, putative	SYA
	MAC_04540	antigenic cell wall galactomannoprotein, putative	SYA, 1/4 SDAY
	MAC_06850	cell wall protein	SYA, 1/4 SDAY
	MAC_05852	antigenic cell wall galactomannoprotein, putative	SYA, 1/4 SDAY
	MAC_00160	cell wall glycosyl hydrolase YteR, putative	SYA, 1/4 SDAY
	MAC_07319	putative cell wall glycoprotein	1/4 SDAY
	MAC_05966	cell wall glycosyl hydrolase Dfg5, putative	SYA, 1/4 SDAY
	MAC_06488	glycine-rich cell wall structural protein 1	1/4 SDAY
	MAC_04903	cell wall galactomannoprotein Mp2/allergen F17-like protein	SYA, 1/4 SDAY
	MAC_01181	cell wall glucanosyltransferase Mwg2	SYA
conidial pigment synthesis	MAC_05385	conidial pigment polyketide synthase PksP/Alb1	SYA, 1/4 SDAY
	MAC_05384	laccase	SYA, 1/4 SDAY
	MAC_04467	laccase Lcc5	SYA
	MAC_02006	laccase Lcc2	1/4 SDAY
conidiation	MAC_03829	APSES transcription factor	SYA
	MAC_02140	UDP-glucose 4-epimerase, putative	SYA, 1/4 SDAY
	MAC_09602	SAM dependent methyltransferase, putative	SYA, 1/4 SDAY
	MAC_08691	developmental protein FluG	SYA
	MAC_03279	methyltransferase LaeA, putative	SYA, 1/4 SDAY
	MAC_07621	sporulation protein RMD8	SYA

Table S4. The possession of genes in central regulatory pathway of conidiation in other fungi. (×: not containing, √: containing, parts: parts of species in the genus

contains.)

	BrlA	AbaA	WetA
Acremonium	×	×	×
Acrodontium	×	×	×
Akanthomyces	×	×	×
Aschersonia	×	×	×
Ascosphaera	×	×	×
Aspergillus	√	√	√
Atkinsiella	×	×	×
Batkoa	×	×	×
Beauveria	×	×	×
Catenaria	×	×	×
Coelomomyces	×	×	×
Conidiobolus	×	×	×
Cordyceps	×	×	×
Culicinomyces	×	×	×
Engyodontium	×	×	×
Entomophthora	×	×	×
Erynia	×	×	×
Eryniopsis	×	×	×
Funicularius	×	×	×
Fusarium	×	√	×
Haplographium	×	×	×
Hirsutella	×	×	×
Hymenostilbe	×	×	×
Hypocrella	×	×	×
Lagenidium	×	×	×
Leptolegnia	×	×	×
Mattirolella	×	×	×
Metarhizium	×	×	×
Microhilum	×	×	×
Myriangium	×	×	×
Nectria	×	×	×
Neozygites	×	×	×
Nomuraea	×	×	×
Paecilomyces	×	×	×
Penicillium	parts	parts	parts
Polycephalomyces	×	×	×
Pseudogibellula	×	×	×
Septobasidium	×	×	×
Smittium	×	×	×
Sorosporella	×	×	×

Sporodiniella	×	×	×
Sporothrix	×	×	×
Sporotrichum	×	×	×
Stilbella	×	×	×
Tilachlidium	×	×	×
Tolypocladium	×	×	×
Torrubiella	×	×	×
Trichothecium	×	×	×
